# Bromide of Potassium and Belladonna in Lythiasis

**Published:** 1872-06

**Authors:** Leonidas Crews

**Affiliations:** Valdosta, Georgia


### BROMIDE OF POTASSIUM AND BELLADONNA IN LITHIASIS.

BY LEONIDAS CREWS, M.D., VALDOSTA, GEORGIA.

In the fall of 1870, I was called to see a gentleman æt. about fifty years, who had been seized very suddenly, a week before, by an attack of *nephritic colic.* He had had his family physician in almost constant attendance from the beginning, and had been plied faithfully with opiates, warm baths, purgatives, diuretics, etc., which afforded nothing more than temporary relief, or an opportunity of resting between the paroxysms of severe pain. Upon my arrival, all other remedies were suspended, and the patient placed immediately on *bromide of potassium, twenty grains, with tincture of belladonna, twenty drops,* to be repeated every three hours. He began to improve at once, and after continuing the prescription twenty-four hours, the paroxysms ceased entirely, and he expressed himself as being well. The medicines were continued, however, for three days (fearing a relapse), the doses being diminished.

Whether the remedy acts as a solvent of renal calculi, or merely promotes their discharge by dilating the ureters, I do not know; but I have used it in several parallel cases, and the result has been the same in each.

In lithiasis, with frequent micturition, ardor urinæ, etc., without renal or cystic calculi, I have repeatedly given the bromide and belladonna during the last four or five years, and can confidently recommend them.

I send you these remarks at the solicitation of Dr. W. A. Love, whom I had the pleasure of meeting a few weeks since. Should they be deemed worthy a place in your JOURNAL, they are at your service.

